# Supplementary material for: Experimentally induced active and quiet sleep engage non-overlapping transcriptional programs in Drosophila
Source: bioRxiv. 2023 Oct 15:2023.04.03.535331. Originally published 2023 Apr 3. Preprint. [Version 3] doi: 10.1101/2023.04.03.535331 (PMC10103959; doi:10.1101/2023.04.03.535331)
Supplement: Supplement 1 — Figure 6-figure supplement 1. Gene Ontology (GO) enrichment analysis for THIP-induced sleep. Significantly downregulated and upregulated GO categories for THIP-sleep (Figure 6-source data 1), listed from most enriched at the top. Broad GO categories are identified below. [file media-1.pdf]

Supplementary Figure 1

	GO Term	Pvalue	Enrichment value
Downregulated	GO:0046460 neutral lipid biosynthetic process	0.0006	52.92
	GO:0046463 acylglycerol biosynthetic process	0.0006	52.92
	GO:0002181 cytoplasmic translation	0.0000	33.41
	GO:0009059 macromolecule biosynthetic process	0.0000	6.44
	GO:0009058 biosynthetic process	0.0000	4.53
	GO:0009064 glutamine family amino acid metabolic process	0.0008	16.54
	GO:0006412 translation	0.0000	11.7
	GO:0043043 peptide biosynthetic process	0.0000	11.54
	GO:0043604 amide biosynthetic process	0.0000	10.48
	GO:0006518 peptide metabolic process	0.0000	8.89
	GO:1901566 organonitrogen compound biosynthetic process	0.0000	6.38
	GO:0044271 cellular nitrogen compound biosynthetic process	0.0000	5.07
	GO:0034641 cellular nitrogen compound metabolic process	0.0000	2.31
	GO:1901564 organonitrogen compound metabolic process	0.0000	2.06
	GO:0006807 nitrogen compound metabolic process	0.0002	1.6
	GO:1901576 organic substance biosynthetic process	0.0000	4.53
	GO:0019752 carboxylic acid metabolic process	0.0008	3.36
	GO:0043436 oxoacid metabolic process	0.0010	3.26
	GO:0006082 organic acid metabolic process	0.0010	3.25
	GO:0019538 protein metabolic process	0.0000	2.27
	GO:0071704 organic substance metabolic process	0.0000	1.9
	GO:0043170 macromolecule metabolic process	0.0001	1.72
	GO:0006591 ornithine metabolic process	0.0006	52.92
	GO:0006525 arginine metabolic process	0.0008	44.1
	GO:1901605 alpha-amino acid metabolic process	0.0003	6.73
	GO:0008152 metabolic process	0.0000	1.83
	GO:0044238 primary metabolic process	0.0000	1.89
	GO:0019432 triglyceride biosynthetic process	0.0003	66.15
	GO:0006414 translational elongation	0.0000	34.51
	GO:0034645 cellular macromolecule biosynthetic process	0.0000	7.55
	GO:0043603 cellular amide metabolic process	0.0000	7.7
	GO:0044249 cellular biosynthetic process	0.0000	4.69
	GO:0044267 cellular protein metabolic process	0.0000	2.58
	GO:0044260 cellular macromolecule metabolic process	0.0000	2.02
	GO:0044237 cellular metabolic process	0.0000	1.67
	GO:0007548 sex differentiation	0.0003	22.05
Upregulated	GO:0006030 chitin metabolic process	0.0003	21.44
	GO:1901071 glucosamine-containing compound metabolic process	0.0004	19.78
	GO:0006040 amino sugar metabolic process	0.0004	19.33
	GO:0006022 aminoglycan metabolic process	0.0006	17.48
	GO:0018990 ecdysis, chitin-based cuticle	0.0002	85.05
	GO:0022404 molting cycle process	0.0004	65.42
	GO:0040003 chitin-based cuticle development	0.0000	32.89
	GO:0042335 cuticle development	0.0000	31.33
	GO:0048856 anatomical structure development	0.0004	4.39

Metabolic process

- **Biosynthetic metabolic process**
- **Nitrogen compound metabolic process**
- **organic substance metabolic process**
- **primary metabolic process**
- **Cellular metabolic process**

Developmental Process

- **Developmental process involved in reproduction**
- **Anatomical structure development**

Mutlicellular organismal process

- **Molting cycle process**
