# Supplementary material for: Experimentally induced active and quiet sleep engage non-overlapping transcriptional programs in Drosophila
Source: bioRxiv. 2023 Oct 15:2023.04.03.535331. Originally published 2023 Apr 3. Preprint. [Version 3] doi: 10.1101/2023.04.03.535331 (PMC10103959; doi:10.1101/2023.04.03.535331)
Supplement: Supplement 2 — Figure 6-figure supplement 2. Gene Ontology (GO) enrichment analysis for THIP-provisioned flies that were sleep deprived. Significantly downregulated and upregulated GO categories for sleep deprived flies (Figure 6-source data 2), listed from most enriched at the top. Broad GO categories are identified below. [file media-2.pdf]

	GO Term	Pvalue	Enrichment value
Downregulated	GO:0104004 cellular response to environmental stimulus	0.0005	19.04
	GO:0050896 response to stimulus	0.0000	3.79
	GO:0034644 cellular response to UV	0.0000	85.05
	GO:0009411 response to UV	0.0001	41.15
	GO:0071482 cellular response to light stimulus	0.0001	32.71
	GO:0071478 cellular response to radiation	0.0003	22.78
	GO:0071214 cellular response to abiotic stimulus	0.0005	19.04
	GO:0009266 response to temperature stimulus	0.0000	16.88
	GO: 0009617 response to bacterium	0.0000	21.07
	GO: 0051707 response to other organism	0.0000	15.39
	GO:0043207 response to external biotic stimulus	0.0000	15.14
	GO: 0009607 response to biotic stimulus	0.0000	15.09
	GO:0050830 defense response to Gram-positive bacterium	0.0000	36.98
	GO:0009605 response to external stimulus	0.0000	9.31
	GO:0034605 cellular response to heat	0.0000	60.75
	GO:0009408 response to heat	0.0000	26.58
	GO:0006979 response to oxidative stress	0.0002	13.83
	GO:0042742 defense response to bacterium	0.0000	12.89
	GO:0098542 defense response to other organism	0.0000	11.63
	GO:0006952 defense response	0.0000	10.12
	GO:0006950 response to stress	0.0000	5.79
	GO:0042381 hemolymph coagulation	0.0001	141.75
	GO:0006955 immune response	0.0007	9.83
	GO:0002376 immune system process	0.0002	9.01
	GO:0007599 hemostasis	0.0001	141.75
	GO:0050878 regulation of body fluid levels	0.0005	56.7
	GO:0051704 multi-organism process	0.0000	12.34
	GO:0050817 coagulation	0.0001	141.75
Upregulated	GO:0008152 metabolic process	0.0000	1.86
	GO:0006032 chitin catabolic process	0.0001	36.6
	GO:1901072 glucosamine-containing compound catabolic process	0.0001	32.53
	GO:0006022 aminoglycan metabolic process	0.0000	13.37
	GO:0006807 nitrogen compound metabolic process	0.0000	1.94
	GO:0046348 amino sugar catabolic process	0.0001	32.53
	GO:0006026 aminoglycan catabolic process	0.0003	21.69
	GO:0006030 chitin metabolic process	0.0000	16.4
	GO:1901071 glucosamine-containing compound metabolic process	0.0000	15.13
	GO:0006040 amino sugar metabolic process	0.0000	14.79
	GO:0006508 proteolysis	0.0000	5.13
	GO:1901135 carbohydrate derivative metabolic process	0.0002	3.94
	GO:1901564 organonitrogen compound metabolic process	0.0000	2.31
	GO:0043170 macromolecule metabolic process	0.0000	2.1
	GO:0071704 organic substance metabolic process	0.0000	1.92
	GO:0019538 protein metabolic process	0.0008	2.01
	GO:0044238 primary metabolic process	0.0004	1.67
	GO:0017144 drug metabolic process	0.0000	6.24

- **Response to stimulus**
  - **Cellular response to stimulus**
  - **Response to abiotic stimulus**
  - **Response to biotic stimulus**
  - **Response to external stimulus**
  - **Response to stress**
- **Mutli-organism process**
  - **Mutliorganism cellular process**
- **MutliCellular organismal process**
  - **Coagulation**
- **Immune system process**
  - **Immune response**
- **Biological regulation**
  - **Regulation of biological quality**
- **Metabolic process**
  - **Metabolic process**
  - **Nitrogen compound metabolic process**
  - **Organic substance**
  - **Primary Metabolic process**
  - **Other**
