# Supplementary figures and images for: Experimentally induced active and quiet sleep engage non-overlapping transcriptional programs in Drosophila
Source: bioRxiv. 2023 Oct 15:2023.04.03.535331. Originally published 2023 Apr 3. Preprint. [Version 3] doi: 10.1101/2023.04.03.535331 (PMC10103959; doi:10.1101/2023.04.03.535331)

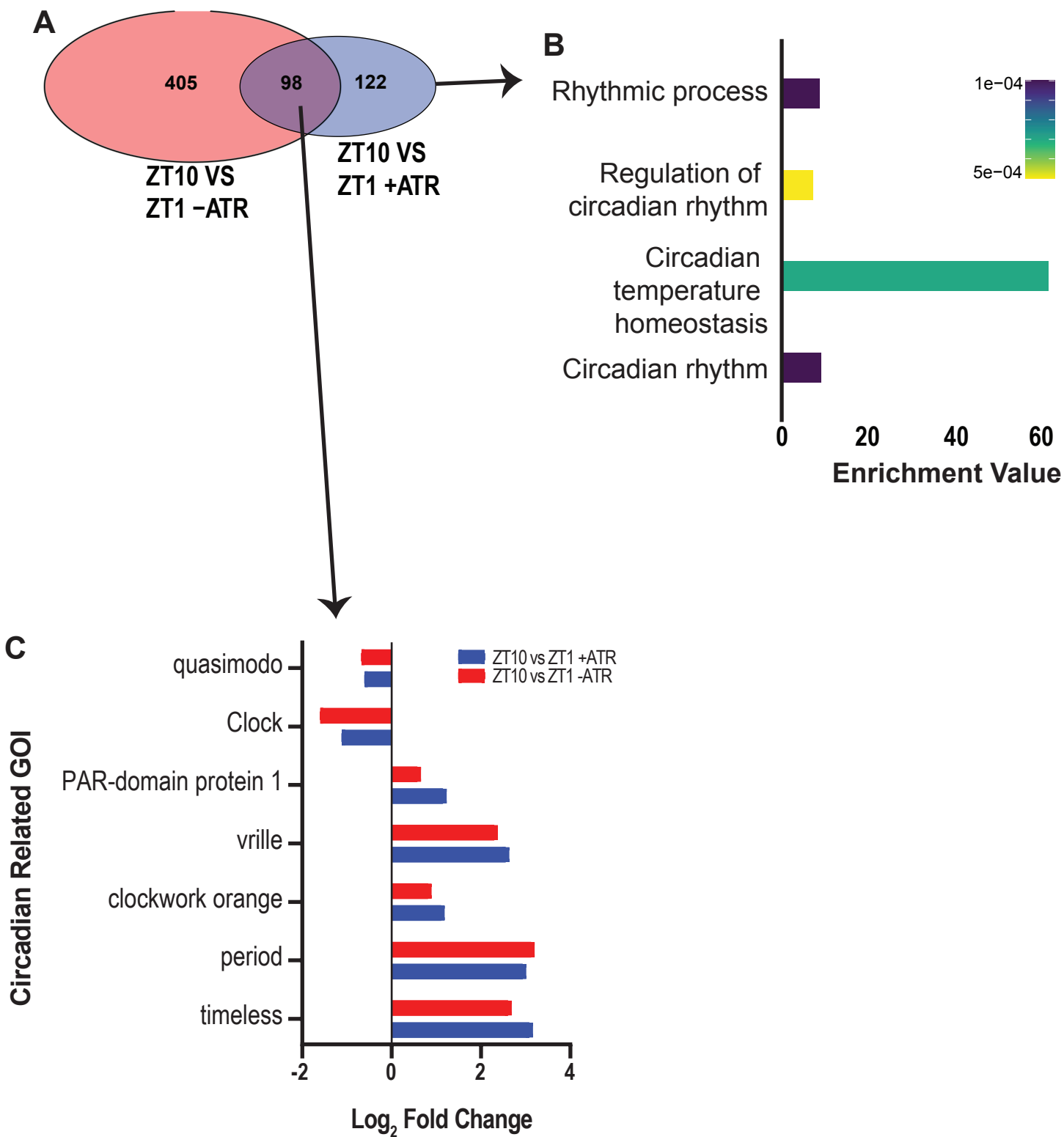

Supplement: Supplement 6 — Figure 7-figure supplement 2. Circadian-related genes uncovered in optogenetic-sleep dataset. A. Zeitgeber (ZT) 10 timepoint was compared with ZT in to uncover potential circadian-regulated genes, in two separate datasets (-ATR and +ATR). 98 genes were shared between these datasets. B. Of the 98 shared genes, circadian-related processes were highly enriched. C. Expression levels of 7 circadian genes drawn from the two different datasets in A. [file media-6.pdf]

# A: Sleep induction method

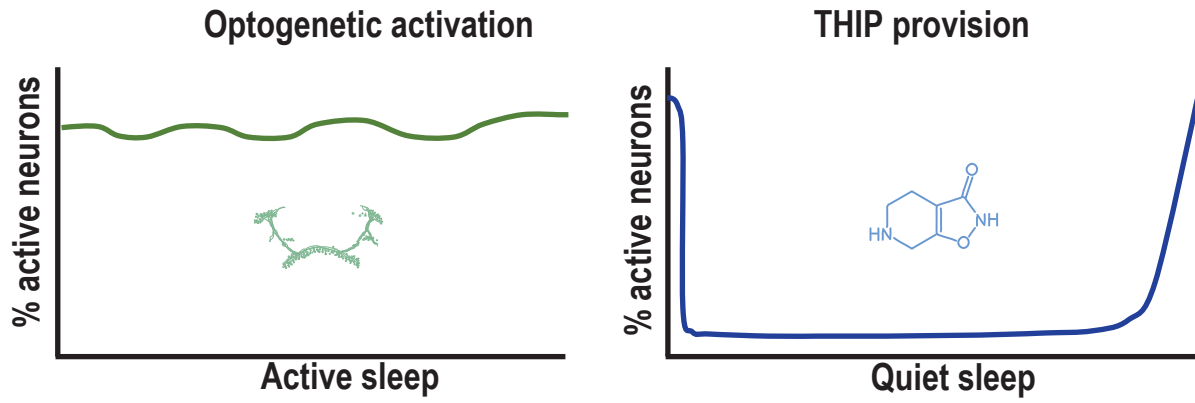

# B: Induced Sleep Transcriptome: GO Pathways of Biological Processes

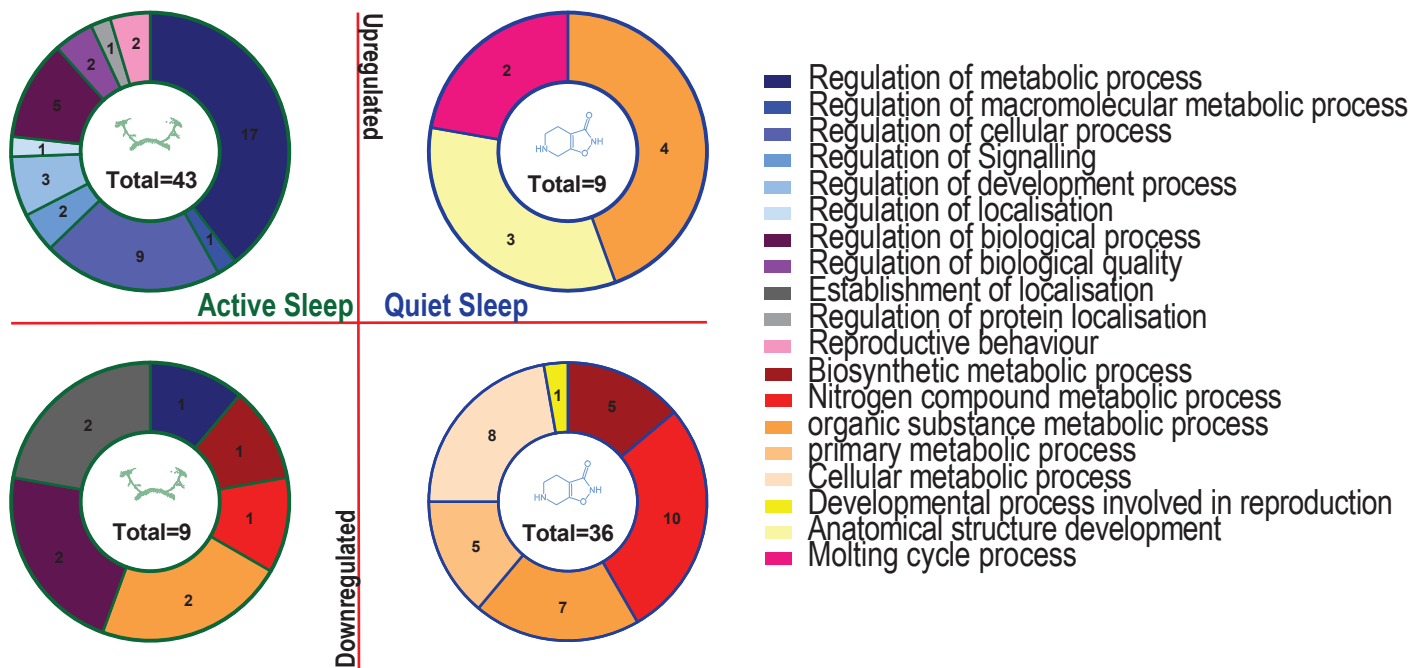

Supplement: Supplement 7 — Figure 7-figure supplement 3. Summary of different Gene Ontogeny pathways engaged by optogenetic-induced sleep and THIP-induced sleep. A. Either sleep induction method produces different levels of activity in the fly brain. We term optogenetic-induced sleep ‘active sleep’ because brain activity levels are not different than during wake. We term THIP-induced sleep ‘quiet sleep’ because neural activity is significant decreased already in the first 5 minutes. Both of these induced forms of sleep resemble sleep stages seen during spontaneous sleep in flies. B. Number of GO pathways engaged by either induced active or quiet sleep, separated by upregulated versus downregulated biological processes. [file media-7.pdf]
