# Supplementary material for: Experimentally induced active and quiet sleep engage non-overlapping transcriptional programs in Drosophila
Source: bioRxiv. 2023 Oct 15:2023.04.03.535331. Originally published 2023 Apr 3. Preprint. [Version 3] doi: 10.1101/2023.04.03.535331 (PMC10103959; doi:10.1101/2023.04.03.535331)
Supplement: Supplement 12 — Figure 8-figure supplement 1. Waking activity levels of nAchRα knockout mutants. Top: mutants are compared to their genetic background strain. Da1–7 = nAchRα1–7. Bottom: statistical tests for waking activity levels in each knockout compared to its genetic control, during both day and night. [file media-12.pdf]

Supplementary Figure 6

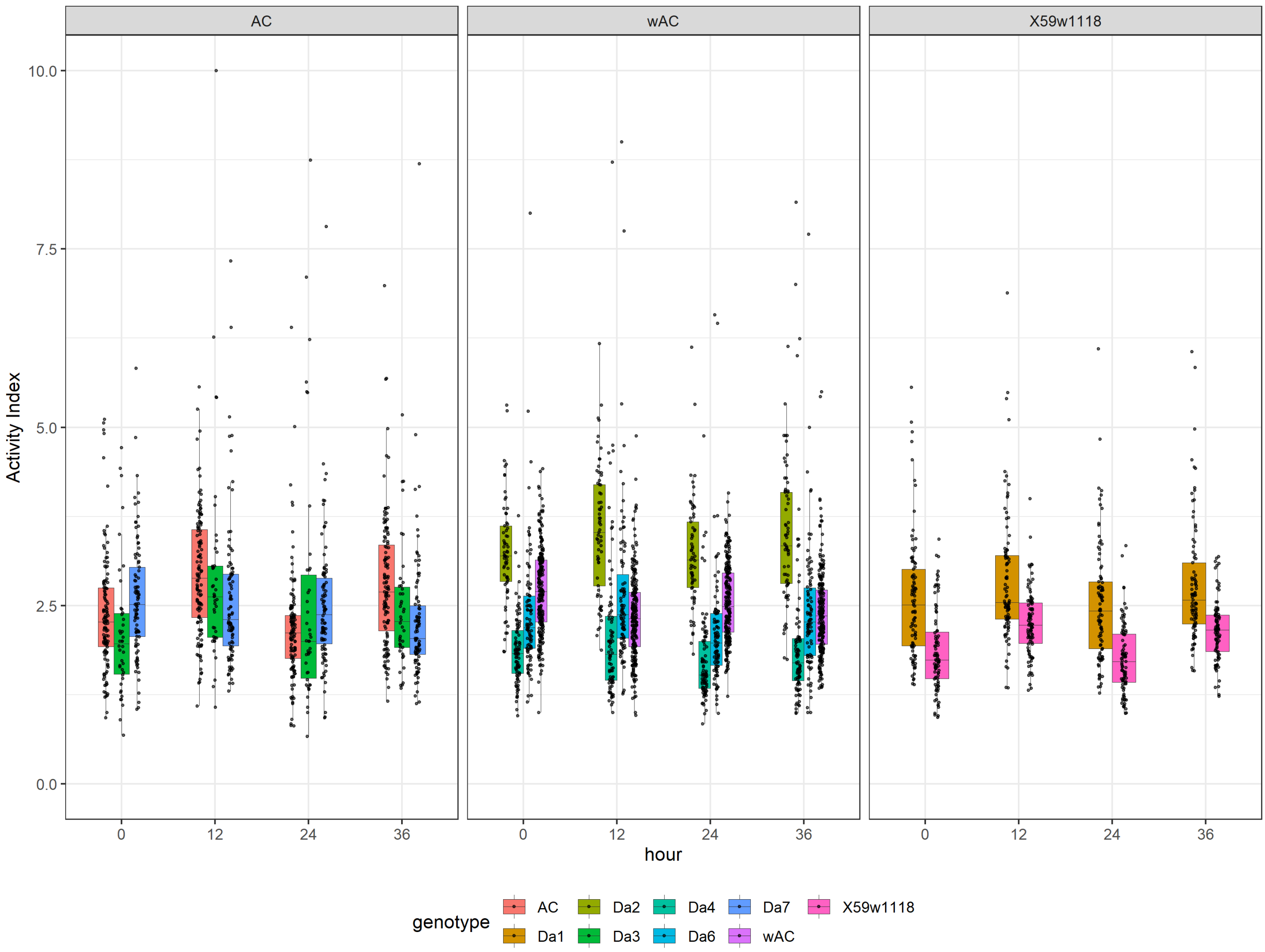

ttest_AI

hour	gene	estimate	estimate1	estimate2	.y.	group1	group2	n1	n2	statistic	p	df	conf.low	conf.high	method	alternative	p.adj	p.adj.signif
**Day**	Da1	0.747011947979923	1.81085006902361	2.55786201700353	activity_index	control	knockout	91	93	-7.68674248159984	1.63e-12	154.732513840803	0.938986358845125	0.555037537114721	T-test	two.sided	1.63e-11	****
**Night**	Da1	0.621402556761431	2.20459173404308	2.82599429080452	activity_index	control	knockout	91	93	-6.52394430852639	1.39e-09	129.928135878636	0.809843317756889	0.432961795765974	T-test	two.sided	9.73e-09	****
**Day**	Da2	0.802741622150127	2.43199942296601	3.23474104511614	activity_index	control	knockout	70	65	-7.4427805261935	2.03e-11	113.734263925721	1.01640704197762	0.589076202322632	T-test	two.sided	1.827e-10	****
**Night**	Da2	1.23901539806697	2.26258176444676	3.50159716251373	activity_index	control	knockout	70	65	-10.1194970663198	8.52e-17	95.6867888875377	1.48206392653957	0.995966869594365	T-test	two.sided	1.1076e-15	****
**Day**	Da3	0.415534948767109	2.16037312206754	2.57590807083465	activity_index	control	knockout	91	43	-1.34323807941607	0.186	45.4132347049576	1.03844775713935	-0.207377859605138	T-test	two.sided	0.744	ns
**Night**	Da3	-0.0227331901857819	2.83159939569465	2.80886620550887	activity_index	control	knockout	91	43	0.0990781987694288	0.921	55.2553143772094	0.43704101471215	-0.482507395083714	T-test	two.sided	1	ns
**Day**	Da4	-0.61837129538045	2.42331822907521	1.80494693369476	activity_index	control	knockout	98	87	8.66914228246151	2.56e-15	178.965595681112	-0.47761492132397	-0.75912766943693	T-test	two.sided	2.816e-14	****
**Night**	Da4	-0.110300614309692	2.26586151382992	2.15556089952023	activity_index	control	knockout	98	87	0.835733550651872	0.405	115.736113589999	0.151109873134961	-0.371711101754344	T-test	two.sided	1	ns
**Day**	Da6	-0.623668327284424	2.88534347323253	2.26167514594811	activity_index	control	knockout	91	91	6.64405236041315	5.29e-10	150.02407366578	-0.43819297313673	-0.809143681432117	T-test	two.sided	4.232e-09	****
**Night**	Da6	0.0488610995058534	2.54149490606688	2.59035600557274	activity_index	control	knockout	91	91	-0.47619750862764	0.635	153.272240702089	0.251567217217754	-0.153845018206047	T-test	two.sided	1	ns
**Day**	Da7	0.326181724510013	2.22741317915403	2.55359490366404	activity_index	control	knockout	95	94	-3.12088657353367	0.00211	176.4794317341	0.532443223747717	0.119920225272308	T-test	two.sided	0.01266	*
**Night**	Da7	-0.236825775153197	2.70854101277	2.4717152376168	activity_index	control	knockout	95	94	1.93009604618506	0.0552	181.674501771687	0.00527760128503382	-0.478929151591428	T-test	two.sided	0.276	ns
