# Supplementary material for: Experimentally induced active and quiet sleep engage non-overlapping transcriptional programs in Drosophila
Source: bioRxiv. 2023 Aug 15:2023.04.03.535331. Originally published 2023 Apr 3. Preprint. [Version 2] doi: 10.1101/2023.04.03.535331 (PMC10103959; doi:10.1101/2023.04.03.535331)
Supplement: Supplementary file 1 [file NIHPP2023.04.03.535331v2-supplement-10.pdf]

	GO Term	Pvalue	Enrichment value
Downregulated	GO:0046460 neutral lipid biosynthetic process	0.0006	52.92
	GO:0046463 acylglycerol biosynthetic process	0.0006	52.92
	GO:0002181 cytoplasmic translation	0.0000	33.41
	GO:0009059 macromolecule biosynthetic process	0.0000	6.44
	GO:0009058 biosynthetic process	0.0000	4.53
	GO:0009064 glutamine family amino acid metabolic process	0.0008	16.54
	GO:0006412 translation	0.0000	11.7
	GO:0043043 peptide biosynthetic process	0.0000	11.54
	GO:0043604 amide biosynthetic process	0.0000	10.48
	GO:0006518 peptide metabolic process	0.0000	8.89
	GO:1901566 organonitrogen compound biosynthetic process	0.0000	6.38
	GO:0044271 cellular nitrogen compound biosynthetic process	0.0000	5.07
	GO:0034641 cellular nitrogen compound metabolic process	0.0000	2.31
	GO:1901564 organonitrogen compound metabolic process	0.0000	2.06
	GO:0006807 nitrogen compound metabolic process	0.0002	1.6
	GO:1901576 organic substance biosynthetic process	0.0000	4.53
	GO:0019752 carboxylic acid metabolic process	0.0008	3.36
	GO:0043436 oxoacid metabolic process	0.0010	3.26
	GO:0006082 organic acid metabolic process	0.0010	3.25
	GO:0019538 protein metabolic process	0.0000	2.27
	GO:0071704 organic substance metabolic process	0.0000	1.9
	GO:0043170 macromolecule metabolic process	0.0001	1.72
	GO:0006591 ornithine metabolic process	0.0006	52.92
	GO:0006525 arginine metabolic process	0.0008	44.1
	GO:1901605 alpha-amino acid metabolic process	0.0003	6.73
	GO:0008152 metabolic process	0.0000	1.83
	GO:0044238 primary metabolic process	0.0000	1.89
	GO:0019432 triglyceride biosynthetic process	0.0003	66.15
	GO:0006414 translational elongation	0.0000	34.51
	GO:0034645 cellular macromolecule biosynthetic process	0.0000	7.55
	GO:0043603 cellular amide metabolic process	0.0000	7.7
	GO:0044249 cellular biosynthetic process	0.0000	4.69
	GO:0044267 cellular protein metabolic process	0.0000	2.58
	GO:0044260 cellular macromolecule metabolic process	0.0000	2.02
	GO:0044237 cellular metabolic process	0.0000	1.67
	GO:0007548 sex differentiation	0.0003	22.05
Upregulated	GO:0006030 chitin metabolic process	0.0003	21.44
	GO:1901071 glucosamine-containing compound metabolic process	0.0004	19.78
	GO:0006040 amino sugar metabolic process	0.0004	19.33
	GO:0006022 aminoglycan metabolic process	0.0006	17.48
	GO:0018990 ecdysis, chitin-based cuticle	0.0002	85.05
	GO:0022404 molting cycle process	0.0004	65.42
	GO:0040003 chitin-based cuticle development	0.0000	32.89
	GO:0042335 cuticle development	0.0000	31.33
	GO:0048856 anatomical structure development	0.0004	4.39

Metabolic process

- **Biosynthetic metabolic process**
- **Nitrogen compound metabolic process**
- **organic substance metabolic process**
- **primary metabolic process**
- **Cellular metabolic process**

Developmental Process

- **Developmental process involved in reproduction**
- **Anatomical structure development**

Mutlicellular organismal process

- **Molting cycle process**

	GO Term	Pvalue	Enrichment value
Downregulated	GO:0104004 cellular response to environmental stimulus	0.0005	19.04
	GO:0050896 response to stimulus	0.0000	3.79
	GO:0034644 cellular response to UV	0.0000	85.05
	GO:0009411 response to UV	0.0001	41.15
	GO:0071482 cellular response to light stimulus	0.0001	32.71
	GO:0071478 cellular response to radiation	0.0003	22.78
	GO:0071214 cellular response to abiotic stimulus	0.0005	19.04
	GO:0009266 response to temperature stimulus	0.0000	16.88
	GO: 0009617 response to bacterium	0.0000	21.07
	GO: 0051707 response to other organism	0.0000	15.39
	GO:0043207 response to external biotic stimulus	0.0000	15.14
	GO: 0009607 response to biotic stimulus	0.0000	15.09
	GO:0050830 defense response to Gram-positive bacterium	0.0000	36.98
	GO:0009605 response to external stimulus	0.0000	9.31
	GO:0034605 cellular response to heat	0.0000	60.75
	GO:0009408 response to heat	0.0000	26.58
	GO:0006979 response to oxidative stress	0.0002	13.83
	GO:0042742 defense response to bacterium	0.0000	12.89
	GO:0098542 defense response to other organism	0.0000	11.63
	GO:0006952 defense response	0.0000	10.12
	GO:0006950 response to stress	0.0000	5.79
	GO:0042381 hemolymph coagulation	0.0001	141.75
	GO:0006955 immune response	0.0007	9.83
	GO:0002376 immune system process	0.0002	9.01
	GO:0007599 hemostasis	0.0001	141.75
	GO:0050878 regulation of body fluid levels	0.0005	56.7
	GO:0051704 multi-organism process	0.0000	12.34
	GO:0050817 coagulation	0.0001	141.75
Upregulated	GO:0008152 metabolic process	0.0000	1.86
	GO:0006032 chitin catabolic process	0.0001	36.6
	GO:1901072 glucosamine-containing compound catabolic process	0.0001	32.53
	GO:0006022 aminoglycan metabolic process	0.0000	13.37
	GO:0006807 nitrogen compound metabolic process	0.0000	1.94
	GO:0046348 amino sugar catabolic process	0.0001	32.53
	GO:0006026 aminoglycan catabolic process	0.0003	21.69
	GO:0006030 chitin metabolic process	0.0000	16.4
	GO:1901071 glucosamine-containing compound metabolic process	0.0000	15.13
	GO:0006040 amino sugar metabolic process	0.0000	14.79
	GO:0006508 proteolysis	0.0000	5.13
	GO:1901135 carbohydrate derivative metabolic process	0.0002	3.94
	GO:1901564 organonitrogen compound metabolic process	0.0000	2.31
	GO:0043170 macromolecule metabolic process	0.0000	2.1
	GO:0071704 organic substance metabolic process	0.0000	1.92
	GO:0019538 protein metabolic process	0.0008	2.01
	GO:0044238 primary metabolic process	0.0004	1.67
	GO:0017144 drug metabolic process	0.0000	6.24

Response to stimulus
- **Cellular response to stimulus**
- **Response to abiotic stimulus**
- **Response to biotic stimulus**
- **Response to external stimulus**
- **Response to stress**

Mutli-organism process
- **Mutliorganism cellular process**

MutliCellular organismal process
- **Coagulation**

Immune system process
- **Immune response**

Biological regulation
- **Regulation of biological quality**

Metabolic process
- **Metabolic process**
- **Nitrogen compound metabolic process**
- **Organic substance**
- **Primary Metabolic process**
- **Other**

	GO Term	Pvalue	Enrichment value
Downregulated	GO:0060148 positive regulation of posttranscriptional gene silencing	0.0005	53.51
	GO:0002181 cytoplasmic translation	0	9.46
	GO:0052803 imidazole-containing compound metabolic process	0.0002	89.19
	GO:0001692 histamine metabolic process	0.0002	89.19
	GO:0042133 neurotransmitter metabolic process	0.0007	16.72
	GO:1900368 regulation of RNA interference	0.0001	133.79
	GO:1900370 positive regulation of RNA interference	0.0001	133.79
	GO:0036466 synaptic vesicle recycling via endosome	0.0003	66.89
	GO:0036465 synaptic vesicle recycling	0.0008	44.6
Upregulated	GO:2000766 negative regulation of cytoplasmic translation	0.0003	63.5
	GO:0017148 negative regulation of translation	0.0002	9.16
	GO:0034249 negative regulation of cellular amide metabolic process	0.0004	7.81
	GO:0006417 regulation of translation	0.0001	5.69
	GO:0034248 regulation of cellular amide metabolic process	0.0001	4.68
	GO:0010608 posttranscriptional regulation of gene expression	0.0001	4.61
	GO:0010468 regulation of gene expression	0	2.21
	GO:0031326 regulation of cellular biosynthetic process	0.0001	2.04
	GO:0009889 regulation of biosynthetic process	0.0001	2.04
	GO:0010556 regulation of macromolecule biosynthetic process	0.0004	1.98
	GO:0060255 regulation of macromolecule metabolic process	0.0001	1.86
	GO:0019222 regulation of metabolic process	0.0001	1.81
	GO:0051171 regulation of nitrogen compound metabolic process	0.0005	1.76
	GO:0080090 regulation of primary metabolic process	0.0006	1.74
	GO:0031323 regulation of cellular metabolic process	0.0007	1.71
	GO:0046011 regulation of oskar mRNA translation	0	29.31
	GO:1902287 semaphorin-plexin signaling pathway involved in axon guidance	0.0001	95.26
	GO:1902285 semaphorin-plexin signaling pathway involved in neuron projection guidance	0.0001	95.26
	GO:0045876 positive regulation of sister chromatid cohesion	0.0001	95.26
	GO:2000305 semaphorin-plexin signaling pathway involved in reg of photoreceptor cell axon guidance	0.0001	95.26
	GO:0048013 ephrin receptor signaling pathway	0.0003	63.5
	GO:0071526 semaphorin-plexin signaling pathway	0	47.63
	GO:0007162 negative regulation of cell adhesion	0.0006	17.86
	GO:0051128 regulation of cellular component organization	0.0002	2.4
	GO:0048522 positive regulation of cellular process	0.0007	1.88
	GO:0099177 regulation of trans-synaptic signaling	0.0001	5.82
	GO:0050804 modulation of chemical synaptic transmission	0.0001	5.82
	GO:0016319 mushroom body development	0.001	6.52
	GO:2000026 regulation of multicellular organismal development	0.0007	2.57
	GO:0032502 developmental process	0.0001	1.81
	GO:0032879 regulation of localization	0.0006	2.72
	GO:2000112 regulation of cellular macromolecule biosynthetic process	0.0004	1.98
	GO:0050794 regulation of cellular process	0	1.84
	GO:0048518 positive regulation of biological process	0.0008	1.8
	GO:0050789 regulation of biological process	0	1.7
	GO:0065007 biological regulation	0	1.63
	GO:0042391 regulation of membrane potential	0.0008	6.9
	GO:0065008 regulation of biological quality	0.0002	2.12
	GO:0120187 positive regulation of protein localization to chromatin	0.0001	95.26
	GO:0008049 male courtship behavior	0.0003	8.36
	GO:0060179 male mating behavior	0.0005	7.56

- **Regulation of biological process**
  - **Regulation of metabolic process**
  - **Regulation of macromolecular metabolic process**
  - **Regulation of cellular process**
  - **Regulation of Signalling**
  - **Regulation of development process**
  - **Regulation of localisation**
- **Metabolic process**
  - **Biosynthetic process**
  - **Nitrogen compound metabolic process**
  - **cellular metabolic process**

- **Biological Regulation**
  - **Regulation of biological process**
  - **Regulation of biological quality**
- **Localisation**
  - **Establishment of localisation**
  - **Regulation of protein localisation**
- **Behaviour**
  - **Reproductive behaviour**

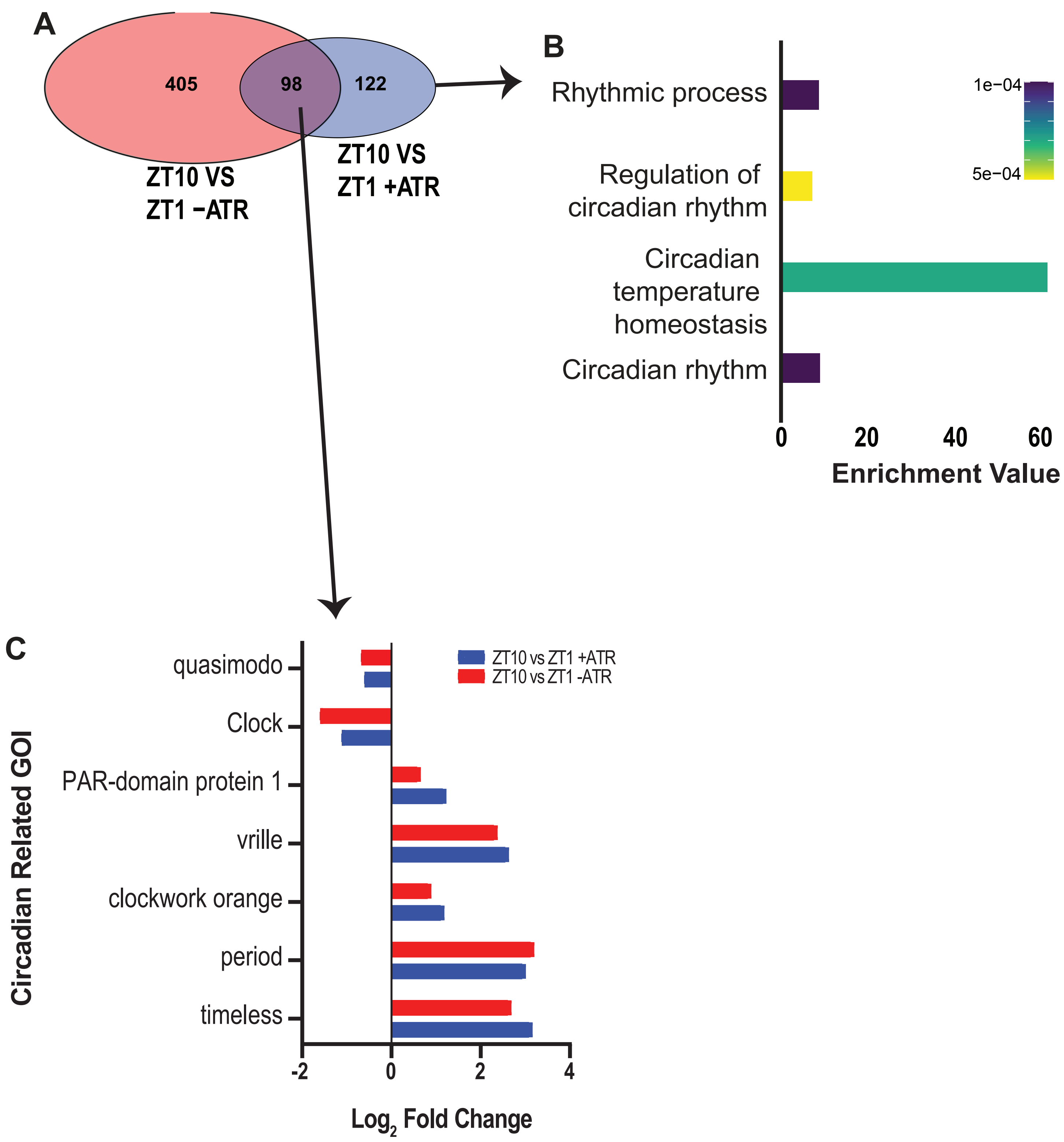
A
405
98
122
ZT10 VS
ZT1 −ATR
ZT10 VS
ZT1 +ATR
B
Rhythmic process
Regulation of circadian rhythm
Circadian temperature homeostasis
Circadian rhythm
1e−04
5e−04
0
20
40
60
Enrichment Value
C
Circadian Related GOI
quasimodo
Clock
PAR-domain protein 1
vrille
clockwork orange
period
timeless
ZT10 vs ZT1 +ATR
ZT10 vs ZT1 -ATR
-2
0
2
4
Log2 Fold Change

Supplementary Figure 5

A: Sleep induction method

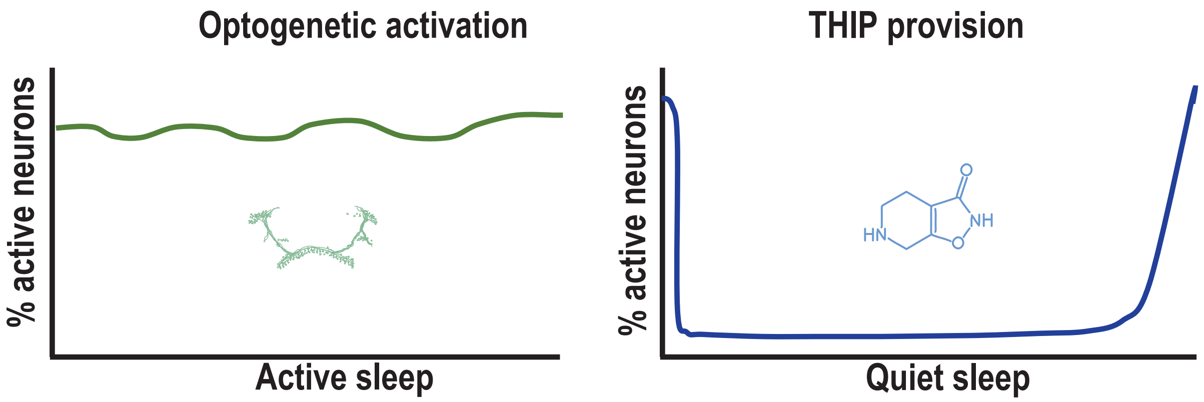

B: Induced Sleep Transcriptome: GO Pathways of Biological Processes

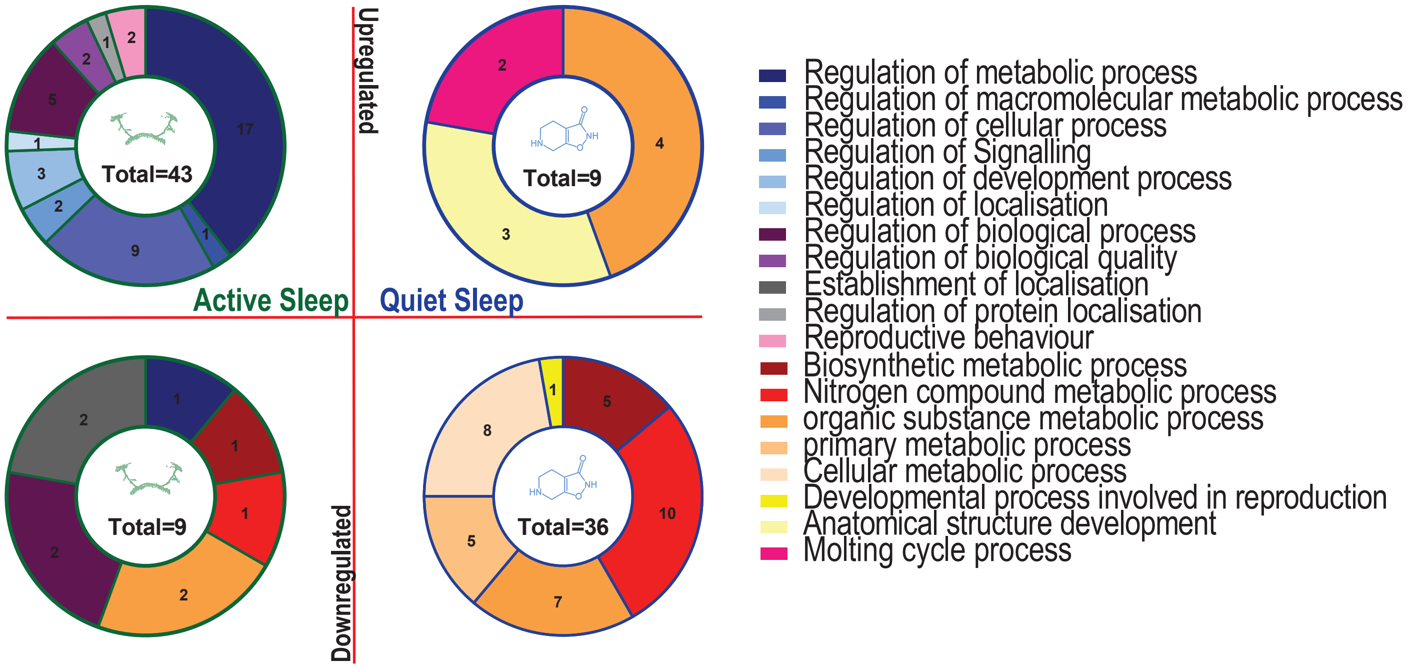

Supplementary Figure 6

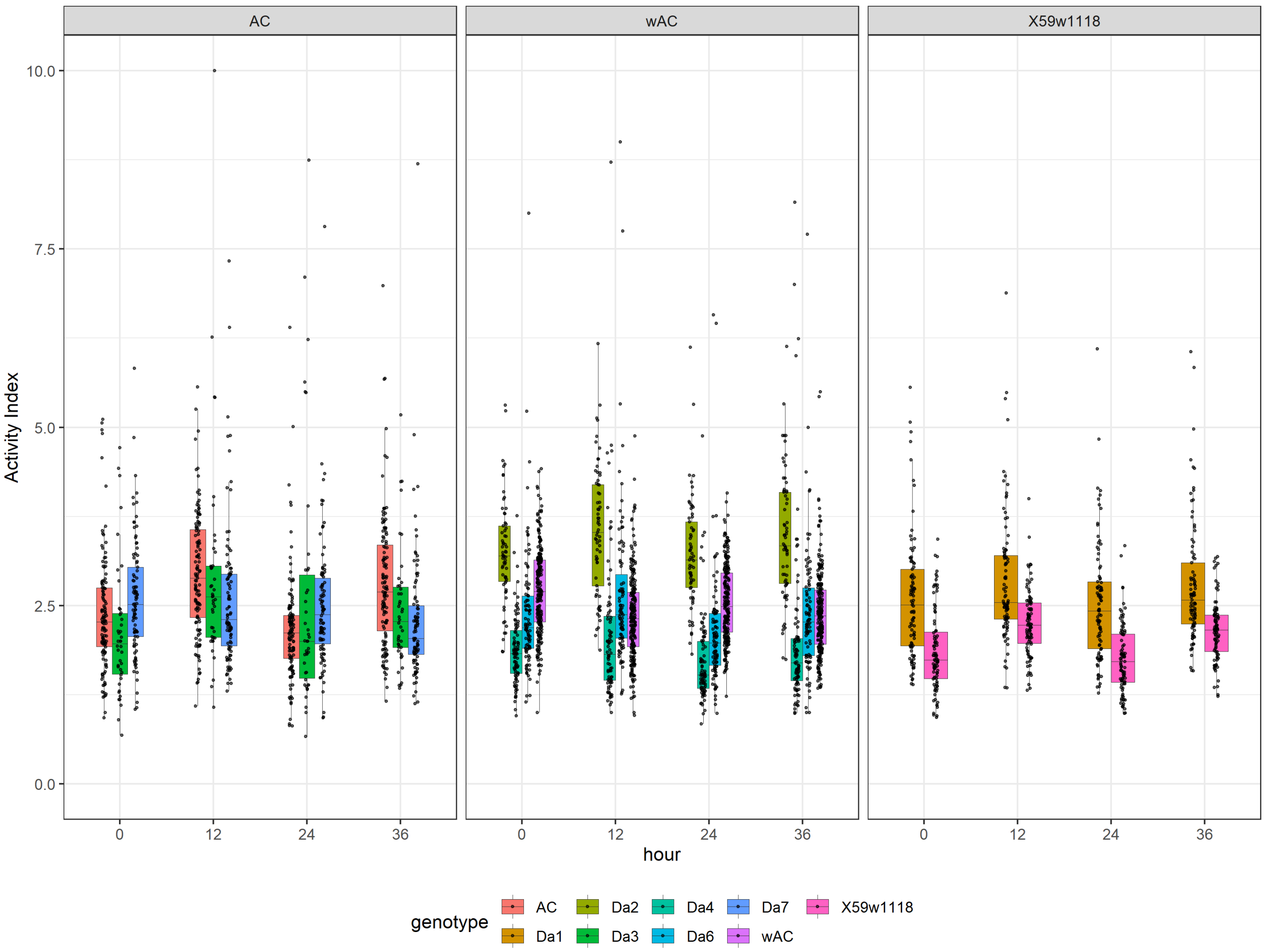

ttest_AI

hour	gene	estimate	estimate1	estimate2	.y.	group1	group2	n1	n2	statistic	p	df	conf.low	conf.high	method	alternative	p.adj	p.adj.signif
**Day**	Da1	0.747011947979923	1.81085006902361	2.55786201700353	activity_index	control	knockout	91	93	-7.68674248159984	1.63e-12	154.732513840803	0.938986358845125	0.555037537114721	T-test	two.sided	1.63e-11	****
**Night**	Da1	0.621402556761431	2.20459173404308	2.82599429080452	activity_index	control	knockout	91	93	-6.52394430852639	1.39e-09	129.928135878636	0.809843317756889	0.432961795765974	T-test	two.sided	9.73e-09	****
**Day**	Da2	0.802741622150127	2.43199942296601	3.23474104511614	activity_index	control	knockout	70	65	-7.4427805261935	2.03e-11	113.734263925721	1.01640704197762	0.589076202322632	T-test	two.sided	1.827e-10	****
**Night**	Da2	1.23901539806697	2.26258176444676	3.50159716251373	activity_index	control	knockout	70	65	-10.1194970663198	8.52e-17	95.6867888875377	1.48206392653957	0.995966869594365	T-test	two.sided	1.1076e-15	****
**Day**	Da3	0.415534948767109	2.16037312206754	2.57590807083465	activity_index	control	knockout	91	43	-1.34323807941607	0.186	45.4132347049576	1.03844775713935	-0.207377859605138	T-test	two.sided	0.744	ns
**Night**	Da3	-0.0227331901857819	2.83159939569465	2.80886620550887	activity_index	control	knockout	91	43	0.0990781987694288	0.921	55.2553143772094	0.43704101471215	-0.482507395083714	T-test	two.sided	1	ns
**Day**	Da4	-0.61837129538045	2.42331822907521	1.80494693369476	activity_index	control	knockout	98	87	8.66914228246151	2.56e-15	178.965595681112	-0.47761492132397	-0.75912766943693	T-test	two.sided	2.816e-14	****
**Night**	Da4	-0.110300614309692	2.26586151382992	2.15556089952023	activity_index	control	knockout	98	87	0.835733550651872	0.405	115.736113589999	0.151109873134961	-0.371711101754344	T-test	two.sided	1	ns
**Day**	Da6	-0.623668327284424	2.88534347323253	2.26167514594811	activity_index	control	knockout	91	91	6.64405236041315	5.29e-10	150.02407366578	-0.43819297313673	-0.809143681432117	T-test	two.sided	4.232e-09	****
**Night**	Da6	0.0488610995058534	2.54149490606688	2.59035600557274	activity_index	control	knockout	91	91	-0.47619750862764	0.635	153.272240702089	0.251567217217754	-0.153845018206047	T-test	two.sided	1	ns
**Day**	Da7	0.326181724510013	2.22741317915403	2.55359490366404	activity_index	control	knockout	95	94	-3.12088657353367	0.00211	176.4794317341	0.532443223747717	0.119920225272308	T-test	two.sided	0.01266	*
**Night**	Da7	-0.236825775153197	2.70854101277	2.4717152376168	activity_index	control	knockout	95	94	1.93009604618506	0.0552	181.674501771687	0.00527760128503382	-0.478929151591428	T-test	two.sided	0.276	ns
