# Supplementary material for: Experimentally induced active and quiet sleep engage non-overlapping transcriptomes in Drosophila
Source: bioRxiv. 2023 Apr 3:2023.04.03.535331. Preprint. [Version 1] doi: 10.1101/2023.04.03.535331 (PMC10103959; doi:10.1101/2023.04.03.535331)

bioRxiv preprint doi: https://doi.org/10.1101/2023.04.03.535331; this version posted April 3, 2023. The copyright holder for this preprint (which was not certified by peer review) is the author/funder, who has granted bioRxiv a license to display the preprint in perpetuity. It is made available under aCC-BY 4.0 International license.

Supplementary Figure Legends

Figure S1. Sleep architecture in dFB and THIP induced sleep (related to Figure 2). **A-B.** Average number of sleep bouts in control (grey) and dFB activation (green) conditions in the day and night for both +ATR **(A)** and –ATR **(B)** fed flies. dFB-induced sleep results in an increase in the number of sleep bouts both during the day and the night, whereas red light alone has no effect. **C-D.** dFB activation (green) increases the average sleep duration during the day, but not the night when compared to controls (grey) in +ATR flies **(C)**. **D.** –ATR flies show no difference in mean sleep bout duration during the day, but show a decrease in average bout duration during the night. THIP (blue) increases both the average number of sleep bouts **(E)** and the average duration of sleep bouts **(F)** during the day, but not the night, when compared to controls (grey). Analysis for a and b = Kruskal-Wallis test with Dunn's multiple comparison correction. * = $p<0.05$, *** = $p < 0.001$, **** = $p<0.0001$. For e and f, analysis = Ordinary one-way ANOVA with Tukey correction for multiple comparisons. *** = $p<0.001$, **** = $p <0.0001$.

Figure S2. Gene Ontology (GO) enrichment analysis for THIP-induced sleep (related to Figure 5). Significantly downregulated and upregulated GO categories for THIP-sleep (Table S2), listed from most enriched at the top. Broad GO categories are identified below.

Figure S3. Gene Ontology (GO) enrichment analysis for THIP-provisioned flies that were sleep deprived (related to Figure 5). Significantly downregulated and upregulated GO categories for sleep deprived flies (Table S3), listed from most enriched at the top. Broad GO categories are identified below.

bioRxiv preprint doi: https://doi.org/10.1101/2023.04.03.535331; this version posted April 3, 2023. The copyright holder for this preprint (which was not certified by peer review) is the author/funder, who has granted bioRxiv a license to display the preprint in perpetuity. It is made available under aCC-BY 4.0 International license.

Figure S4. Gene Ontology (GO) enrichment analysis for dFB-induced sleep (related to Figure 7). Significantly downregulated and upregulated GO categories for dFB-sleep (Table S4), listed from most enriched at the top. Broad GO categories are identified below.

Figure S5. Circadian-related genes uncovered in dFB-sleep dataset (related to Figure 7). **A.** Zeitgeber (ZT) 10 timepoint was compared with ZT in to uncover potential circadian-regulated genes, in two separate datasets (-ATR and +ATR). 98 genes were shared between these datasets. **B.** Of the 98 shared genes, circadian-related processes were highly enriched. **C.** Expression levels of 7 circadian genes drawn from the two different datasets in A.

Figure S6. Summary of different Gene Ontogeny pathways engaged by dFB-induced sleep and THIP-induced sleep. A. Either sleep induction method produces different levels of activity in the fly brain. WE term dFB-induced sleep ‘active sleep’ because brain activity levels are not different than during wake. We term THIP-induced sleep ‘quiet sleep’ because neural activity is significant decreased already in the first 5 minutes. Both of these induced forms of sleep resemble sleep stages seen during spontaneous sleep in flies. **B.** Number of GO pathways engaged by either induced active or quiet sleep, separated by upregulated versus downregulated biological processes.

bioRxiv preprint doi: https://doi.org/10.1101/2023.04.03.535331; this version posted April 3, 2023. The copyright holder for this preprint (which was not certified by peer review) is the author/funder, who has granted bioRxiv a license to display the preprint in perpetuity. It is made available under aCC-BY 4.0 International license.

Supplementary Tables

Table S1, related to Figure 2. A comparison of sleep duration profiles (min/hr) during dFB and THIP induced sleep. Tested with 2way ANOVA with Tukey's multiple comparison test.

Table S2, related to Figure 5. List of significant THIP-sleep genes.

Table S3, related to Figure 5. List of significant sleep-deprivation genes in THIP-fed flies.

Table S4, related to Figure 6. List of significant dFB-sleep genes after 10 hours activation.

Table S5, related to Figure 6. List of significant dFB-sleep genes after 1 hour of activation.

Table S6, related to Figure 6. List of significant ZT10 vs ZT1 genes in ATR+ dataset.

Table S7, related to Figure 6. List of significant ZT10 vs ZT1 genes in ATR- dataset.

Table S8, related to Figures 5 & 6. Primer list for RT qPCR validation experiments.

bioRxiv preprint doi: https://doi.org/10.1101/2023.04.03.535331; this version posted April 3, 2023. The copyright holder for this preprint (which was not certified by peer review) is the author/funder, who has granted bioRxiv a license to display the preprint in perpetuity. It is made available under aCC-BY 4.0 International license.

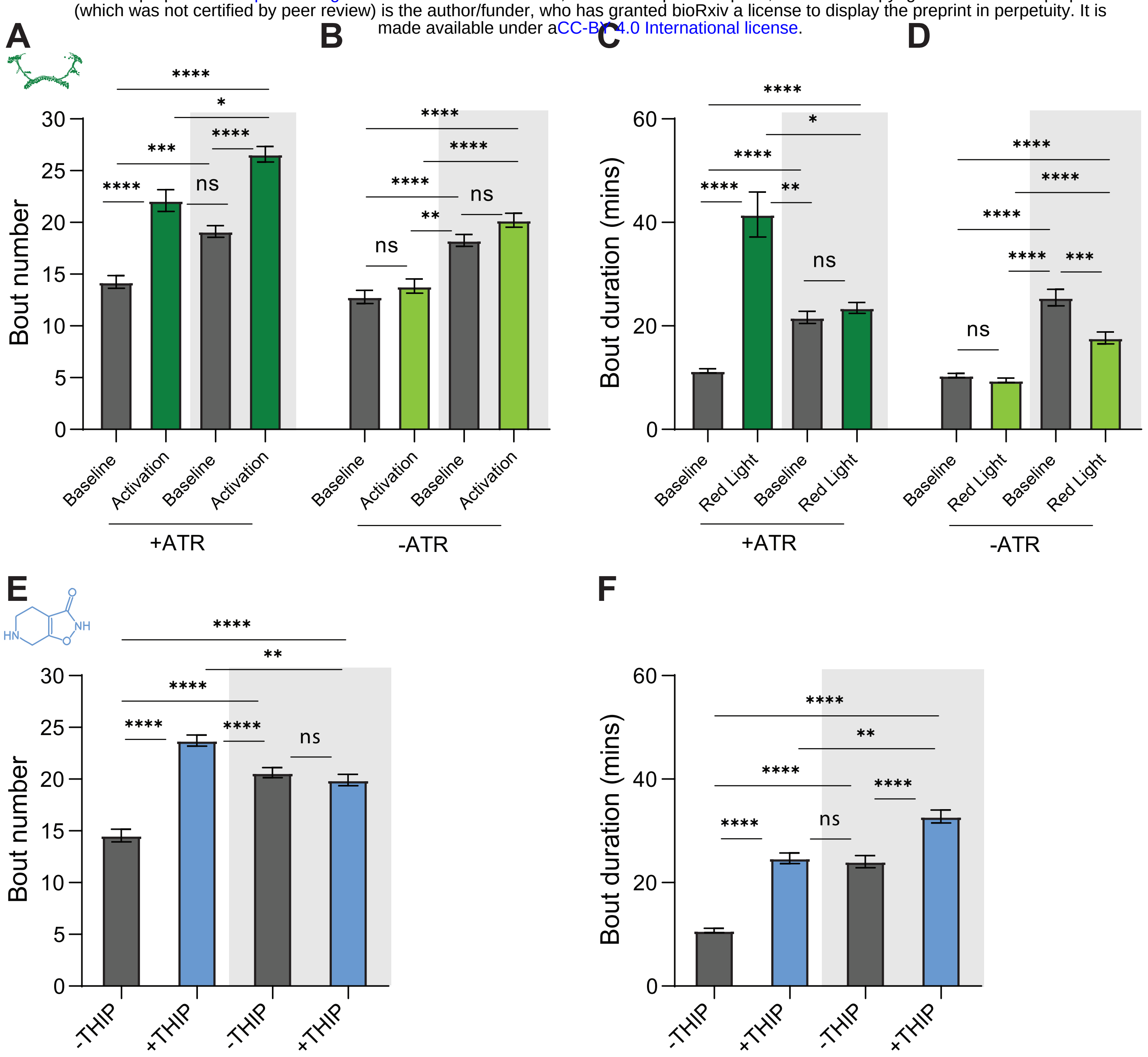

bioRxiv preprint doi: https://doi.org/10.1101/2023.04.03.535331; this version posted April 3, 2023. The copyright holder for this preprint (which was not certified by peer review) is the author/funder, who has granted bioRxiv a license to display the preprint in perpetuity. It is made available under aCC-BY 4.0 International license.

Supplementary Figure 2

	GO Term	Pvalue	Enrichment value
Downregulated	GO:0046460 neutral lipid biosynthetic process	0.0006	52.92
	GO:0046463 acylglycerol biosynthetic process	0.0006	52.92
	GO:0002181 cytoplasmic translation	0.0000	33.41
	GO:0009059 macromolecule biosynthetic process	0.0000	6.44
	GO:0009058 biosynthetic process	0.0000	4.53
	GO:0009064 glutamine family amino acid metabolic process	0.0008	16.54
	GO:0006412 translation	0.0000	11.7
	GO:0043043 peptide biosynthetic process	0.0000	11.54
	GO:0043604 amide biosynthetic process	0.0000	10.48
	GO:0006518 peptide metabolic process	0.0000	8.89
	GO:1901566 organonitrogen compound biosynthetic process	0.0000	6.38
	GO:0044271 cellular nitrogen compound biosynthetic process	0.0000	5.07
	GO:0034641 cellular nitrogen compound metabolic process	0.0000	2.31
	GO:1901564 organonitrogen compound metabolic process	0.0000	2.06
	GO:0006807 nitrogen compound metabolic process	0.0002	1.6
	GO:1901576 organic substance biosynthetic process	0.0000	4.53
	GO:0019752 carboxylic acid metabolic process	0.0008	3.36
	GO:0043436 oxoacid metabolic process	0.0010	3.26
	GO:0006082 organic acid metabolic process	0.0010	3.25
	GO:0019538 protein metabolic process	0.0000	2.27
	GO:0071704 organic substance metabolic process	0.0000	1.9
	GO:0043170 macromolecule metabolic process	0.0001	1.72
	GO:0006591 ornithine metabolic process	0.0006	52.92
	GO:0006525 arginine metabolic process	0.0008	44.1
	GO:1901605 alpha-amino acid metabolic process	0.0003	6.73
	GO:0008152 metabolic process	0.0000	1.83
	GO:0044238 primary metabolic process	0.0000	1.89
	GO:0019432 triglyceride biosynthetic process	0.0003	66.15
	GO:0006414 translational elongation	0.0000	34.51
	GO:0034645 cellular macromolecule biosynthetic process	0.0000	7.55
	GO:0043603 cellular amide metabolic process	0.0000	7.7
	GO:0044249 cellular biosynthetic process	0.0000	4.69
	GO:0044267 cellular protein metabolic process	0.0000	2.58
	GO:0044260 cellular macromolecule metabolic process	0.0000	2.02
	GO:0044237 cellular metabolic process	0.0000	1.67
	GO:0007548 sex differentiation	0.0003	22.05
Upregulated	GO:0006030 chitin metabolic process	0.0003	21.44
	GO:1901071 glucosamine-containing compound metabolic process	0.0004	19.78
	GO:0006040 amino sugar metabolic process	0.0004	19.33
	GO:0006022 aminoglycan metabolic process	0.0006	17.48
	GO:0018990 ecdysis, chitin-based cuticle	0.0002	85.05
	GO:0022404 molting cycle process	0.0004	65.42
	GO:0040003 chitin-based cuticle development	0.0000	32.89
	GO:0042335 cuticle development	0.0000	31.33
	GO:0048856 anatomical structure development	0.0004	4.39

Metabolic process

- **Biosynthetic metabolic process**
- **Nitrogen compound metabolic process**
- **organic substance metabolic process**
- **primary metabolic process**
- **Cellular metabolic process**

Developmental Process

- **Developmental process involved in reproduction**
- **Anatomical structure development**

Mutlicellular organismal process

- **Molting cycle process**

bioRxiv preprint doi: https://doi.org/10.1101/2023.04.03.535331; this version posted April 3, 2023. The copyright holder for this preprint (which was not certified by peer review) is the author/funder, who has granted bioRxiv a license to display the preprint in perpetuity. It is made available under aCC-BY 4.0 International license.

Supplementary Figure 3

	GO Term	Pvalue	Enrichment value
Downregulated	GO:0104004 cellular response to environmental stimulus	0.0005	19.04
	GO:0050896 response to stimulus	0.0000	3.79
	GO:0034644 cellular response to UV	0.0000	85.05
	GO:0009411 response to UV	0.0001	41.15
	GO:0071482 cellular response to light stimulus	0.0001	32.71
	GO:0071478 cellular response to radiation	0.0003	22.78
	GO:0071214 cellular response to abiotic stimulus	0.0005	19.04
	GO:0009266 response to temperature stimulus	0.0000	16.88
	GO: 0009617 response to bacterium	0.0000	21.07
	GO: 0051707 response to other organism	0.0000	15.39
	GO:0043207 response to external biotic stimulus	0.0000	15.14
	GO: 0009607 response to biotic stimulus	0.0000	15.09
	GO:0050830 defense response to Gram-positive bacterium	0.0000	36.98
	GO:0009605 response to external stimulus	0.0000	9.31
	GO:0034605 cellular response to heat	0.0000	60.75
	GO:0009408 response to heat	0.0000	26.58
	GO:0006979 response to oxidative stress	0.0002	13.83
	GO:0042742 defense response to bacterium	0.0000	12.89
	GO:0098542 defense response to other organism	0.0000	11.63
	GO:0006952 defense response	0.0000	10.12
	GO:0006950 response to stress	0.0000	5.79
	GO:0042381 hemolymph coagulation	0.0001	141.75
	GO:0006955 immune response	0.0007	9.83
	GO:0002376 immune system process	0.0002	9.01
	GO:0007599 hemostasis	0.0001	141.75
	GO:0050878 regulation of body fluid levels	0.0005	56.7
	GO:0051704 multi-organism process	0.0000	12.34
	GO:0050817 coagulation	0.0001	141.75
Upregulated	GO:0008152 metabolic process	0.0000	1.86
	GO:0006032 chitin catabolic process	0.0001	36.6
	GO:1901072 glucosamine-containing compound catabolic process	0.0001	32.53
	GO:0006022 aminoglycan metabolic process	0.0000	13.37
	GO:0006807 nitrogen compound metabolic process	0.0000	1.94
	GO:0046348 amino sugar catabolic process	0.0001	32.53
	GO:0006026 aminoglycan catabolic process	0.0003	21.69
	GO:0006030 chitin metabolic process	0.0000	16.4
	GO:1901071 glucosamine-containing compound metabolic process	0.0000	15.13
	GO:0006040 amino sugar metabolic process	0.0000	14.79
	GO:0006508 proteolysis	0.0000	5.13
	GO:1901135 carbohydrate derivative metabolic process	0.0002	3.94
	GO:1901564 organonitrogen compound metabolic process	0.0000	2.31
	GO:0043170 macromolecule metabolic process	0.0000	2.1
	GO:0071704 organic substance metabolic process	0.0000	1.92
	GO:0019538 protein metabolic process	0.0008	2.01
	GO:0044238 primary metabolic process	0.0004	1.67
	GO:0017144 drug metabolic process	0.0000	6.24

Response to stimulus
- **Cellular response to stimulus**
- **Response to abiotic stimulus**
- **Response to biotic stimulus**
- **Response to external stimulus**
- **Response to stress**

Mutli-organism process
- **Mutliorganism cellular process**

MutliCellular organismal process
- **Coagulation**

Immune system process
- **Immune response**

Biological regulation
- **Regulation of biological quality**

Metabolic process
- **Metabolic process**
- **Nitrogen compound metabolic process**
- **Organic substance**
- **Primary Metabolic process**
- **Other**

bioRxiv preprint doi: https://doi.org/10.1101/2023.04.03.535331; this version posted April 3, 2023. The copyright holder for this preprint (which was not certified by peer review) is the author/funder, who has granted bioRxiv a license to display the preprint in perpetuity. It is made available under aCC-BY 4.0 International license.

Supplementary Figure 4

	GO Term	Pvalue	Enrichment value
Downregulated	GO:0060148 positive regulation of posttranscriptional gene silencing	0.0005	53.51
	GO:0002181 cytoplasmic translation	0	9.46
	GO:0052803 imidazole-containing compound metabolic process	0.0002	89.19
	GO:0001692 histamine metabolic process	0.0002	89.19
	GO:0042133 neurotransmitter metabolic process	0.0007	16.72
	GO:1900368 regulation of RNA interference	0.0001	133.79
	GO:1900370 positive regulation of RNA interference	0.0001	133.79
	GO:0036466 synaptic vesicle recycling via endosome	0.0003	66.89
	GO:0036465 synaptic vesicle recycling	0.0008	44.6
Upregulated	GO:2000766 negative regulation of cytoplasmic translation	0.0003	63.5
	GO:0017148 negative regulation of translation	0.0002	9.16
	GO:0034249 negative regulation of cellular amide metabolic process	0.0004	7.81
	GO:0006417 regulation of translation	0.0001	5.69
	GO:0034248 regulation of cellular amide metabolic process	0.0001	4.68
	GO:0010608 posttranscriptional regulation of gene expression	0.0001	4.61
	GO:0010468 regulation of gene expression	0	2.21
	GO:0031326 regulation of cellular biosynthetic process	0.0001	2.04
	GO:0009889 regulation of biosynthetic process	0.0001	2.04
	GO:0010556 regulation of macromolecule biosynthetic process	0.0004	1.98
	GO:0060255 regulation of macromolecule metabolic process	0.0001	1.86
	GO:0019222 regulation of metabolic process	0.0001	1.81
	GO:0051171 regulation of nitrogen compound metabolic process	0.0005	1.76
	GO:0080090 regulation of primary metabolic process	0.0006	1.74
	GO:0031323 regulation of cellular metabolic process	0.0007	1.71
	GO:0046011 regulation of oskar mRNA translation	0	29.31
	GO:1902287 semaphorin-plexin signaling pathway involved in axon guidance	0.0001	95.26
	GO:1902285 semaphorin-plexin signaling pathway involved in neuron projection guidance	0.0001	95.26
	GO:0045876 positive regulation of sister chromatid cohesion	0.0001	95.26
	GO:2000305 semaphorin-plexin signaling pathway involved in reg of photoreceptor cell axon guidance	0.0001	95.26
	GO:0048013 ephrin receptor signaling pathway	0.0003	63.5
	GO:0071526 semaphorin-plexin signaling pathway	0	47.63
	GO:0007162 negative regulation of cell adhesion	0.0006	17.86
	GO:0051128 regulation of cellular component organization	0.0002	2.4
	GO:0048522 positive regulation of cellular process	0.0007	1.88
	GO:0099177 regulation of trans-synaptic signaling	0.0001	5.82
	GO:0050804 modulation of chemical synaptic transmission	0.0001	5.82
	GO:0016319 mushroom body development	0.001	6.52
	GO:2000026 regulation of multicellular organismal development	0.0007	2.57
	GO:0032502 developmental process	0.0001	1.81
	GO:0032879 regulation of localization	0.0006	2.72
	GO:2000112 regulation of cellular macromolecule biosynthetic process	0.0004	1.98
	GO:0050794 regulation of cellular process	0	1.84
	GO:0048518 positive regulation of biological process	0.0008	1.8
	GO:0050789 regulation of biological process	0	1.7
	GO:0065007 biological regulation	0	1.63
	GO:0042391 regulation of membrane potential	0.0008	6.9
	GO:0065008 regulation of biological quality	0.0002	2.12
	GO:0120187 positive regulation of protein localization to chromatin	0.0001	95.26
	GO:0008049 male courtship behavior	0.0003	8.36
	GO:0060179 male mating behavior	0.0005	7.56

Regulation of biological process
- **Regulation of metabolic process**
- **Regulation of macromolecular metabolic process**
- **Regulation of cellular process**
- **Regulation of Signalling**
- **Regulation of development process**
- **Regulation of localisation**

Metabolic process
- **Biosynthetic process**
- **Nitrogen compound metabolic process**
- **cellular metabolic process**

Biological Regulation
- **Regulation of biological process**
- **Regulation of biological quality**

Localisation
- **Establishment of localisation**
- **Regulation of protein localisation**

Behaviour
- **Reproductive behaviour**

bioRxiv preprint doi: https://doi.org/10.1101/2023.04.03.535331; this version posted April 3, 2023. The copyright holder for this preprint (which was not certified by peer review) is the author/funder, who has granted bioRxiv a license to display the preprint in perpetuity. It is made available under aCC-BY 4.0 International license.

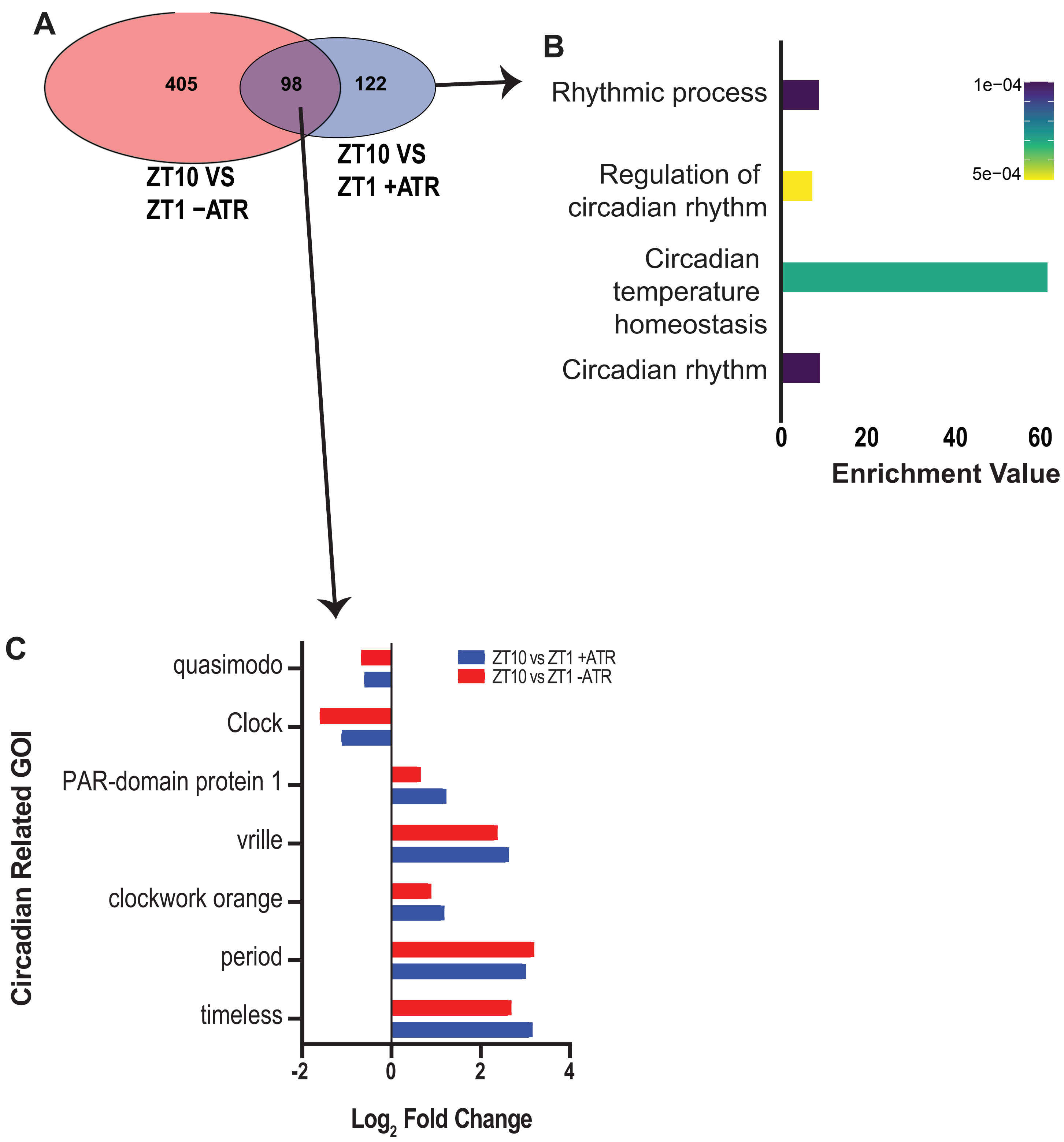

bioRxiv preprint doi: https://doi.org/10.1101/2023.04.03.535331; this version posted April 3, 2023. The copyright holder for this preprint (which was not certified by peer review) is the author/funder, who has granted bioRxiv a license to display the preprint in perpetuity. It is made available under aCC-BY 4.0 International license.

Supplementary Figure 6

A: Sleep induction method

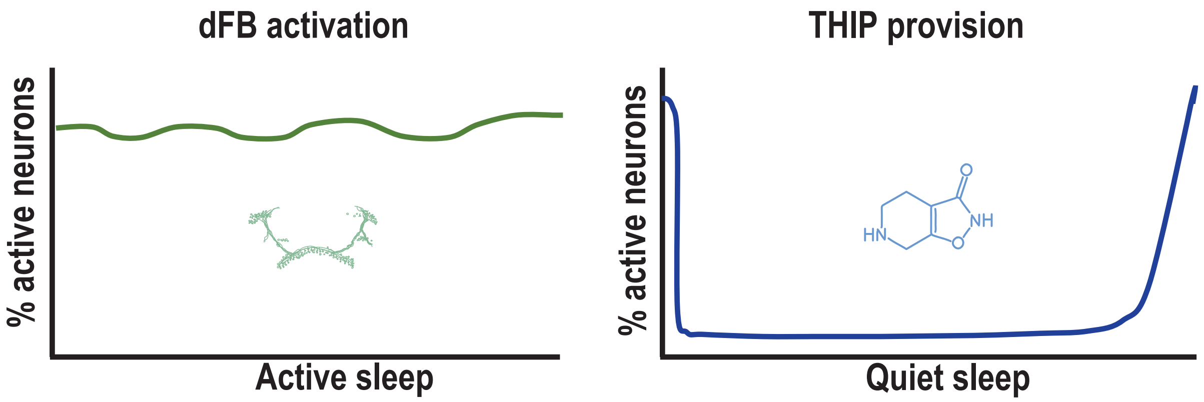

B: Induced Sleep Transcriptome: GO Pathways of Biological Processes

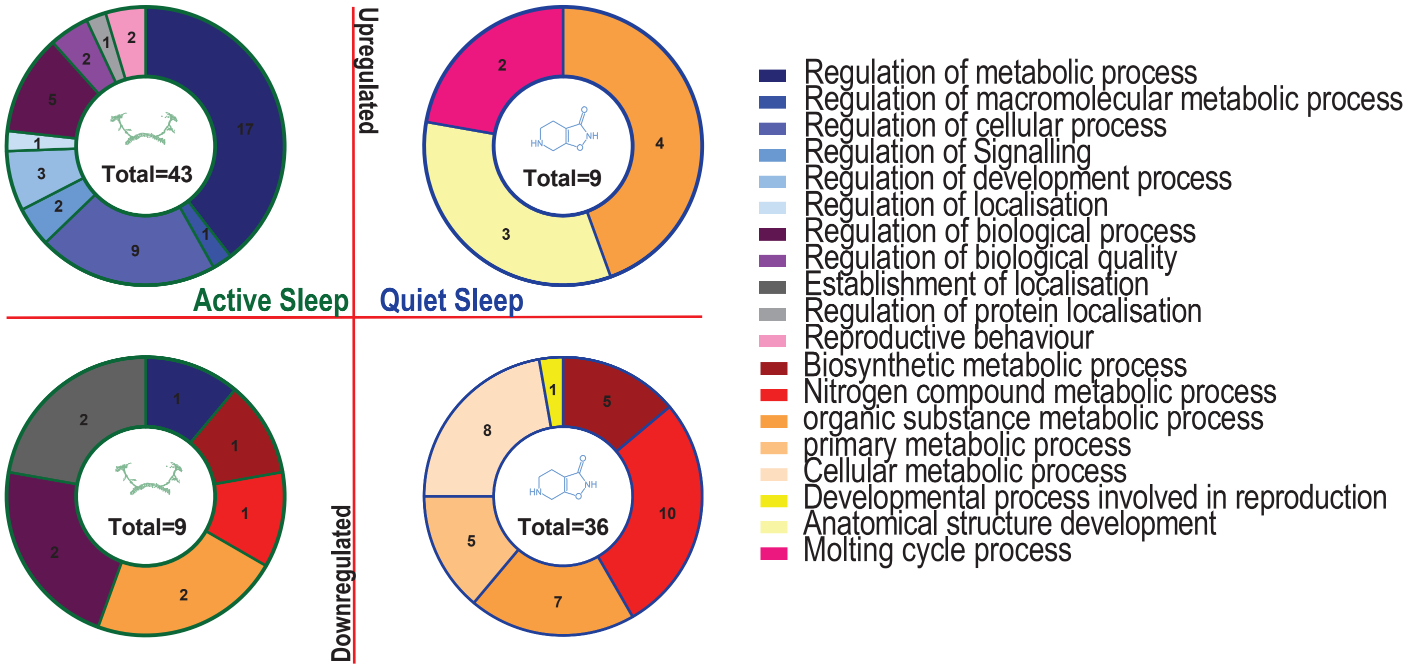

Supplement: Supplementary file 1 [file NIHPP2023.04.03.535331v1-supplement-1.pdf]
